# Interference of stress with the somatotropic axis in pigs – lights on new biomarkers

**DOI:** 10.1038/s41598-017-11521-5

**Published:** 2017-09-21

**Authors:** Elisa Wirthgen, Martin Kunze, Sébastien Goumon, Christina Walz, Christine Höflich, Marion Spitschak, Julia Brenmoehl, Ellen Kanitz, Margret Tuchscherer, Winfried Otten, Ulrike Gimsa, Peter Schön, Christian Manteuffel, Armin Tuchscherer, Ralf Pfuhl, Cornelia C. Metges, Bernd Stabenow, Sandra Erdmann, Kathleen Schluricke, Luigi Faucitano, Andreas Hoeflich

**Affiliations:** 1 Institute of Genome Biology, Leibniz Institute for Farm Animal Biology (FBN), Dummerstorf, Germany; 2 Institute of Animal Science, Department of Ethology, Prague, Czech Republic; 3 Ligandis GbR, 18276 Gülzow-Prüzen, Germany; 4 Institute of Behavioural Physiology, Leibniz Institute for Farm Animal Biology (FBN), Dummerstorf, Germany; 5 Institute of Genetics and Biometry, Leibniz Institute for Farm Animal Biology (FBN), Dummerstorf, Germany; 6 Institute of Muscle Biology & Growth, Leibniz Institute for Farm Animal Biology (FBN), Dummerstorf, Germany; 7 Institute of Nutritional Physiology ‘Oskar Kellner’, Leibniz Institute for Farm Animal Biology (FBN), Dummerstorf, Germany; 8 Experimental Animal Facilities, Leibniz Institute for Farm Animal Biology (FBN), Dummerstorf, Germany; 9 Fleischwerk EDEKA Nord GmbH, 19246 Lüttow-Valluhn, Germany; 10 Sherbrooke Research and Development Centre, Sherbrooke, Canada

## Abstract

The acceptance of animal products is increasingly associated with standardized animal welfare, which relates to appropriate animal husbandry from birth to slaughter. In particular, shipment to the slaughterhouse is considered as a critical process exposing the animals to a number of, in part severe, stressors. New biomarkers may be useful for the assessment of animal welfare. The IGF-system has been assessed in a commercial pig transport in conjunction with established markers of stress response. Furthermore, the effect of repeated restraint as an experimental model for repeated acute stress was investigated. During shipment from farm to slaughterhouse, plasma concentrations of IGFBP-3 and IGFBP-2 were significantly reduced (p < 0.01). After shipment, the plasma concentrations of IGFBP-5, glucocorticoids and IL-2 increased but decreased after lairage (p < 0.05) whereas IGF-1 decreased after shipment (p < 0.01). Repeated acute stress increased concentrations of IGFBP-3 and IGF-1 in exsanguination blood (p < 0.05). Differential IGF- signatures can indicate altered endocrine or metabolic control and thus contain complex animal-related information. The somatotropic axis may be of particular interest when established biomarkers such as cortisol, glucose, or lactate cannot be used for the assessment of animal stress or welfare.

## Introduction

Industrialized production of pork is an important element of the agriculture sector with high economic relevance, which results in pork being the most popular meat product worldwide^1^. Due to the specialization of pork production chain into different segments, there has been an increased need to transport large numbers of pigs, e.g. from the nursery to the growing and/or grow-to-finish farm and eventually to the slaughterhouse. There is an overall agreement that the transportation event includes multifaceted psychological and physical stress factors, which can have detrimental effects on the health and welfare of farm animals^2–5^ resulting in the concurrent activation of endocrine, metabolic or immune pathways^6^. In response to the increasing public demand for the respect of animal welfare in the livestock production practices, the identification and validation of objective biomarkers for a standardized monitoring of the health and welfare of livestock are needed. However, the value of the assessment of stress-associated blood parameters, such as glucocorticoids, catecholamines or glucose, is often limited due to the interference of different stress factors, genotype-specific stress reactions or poor practical feasibility^7^. Furthermore, it has been shown that repeated short- or long-term stress (i.e. tail biting) can result in a blunted stress response of the HPA-axis in rats and pigs^8–10^. With respect to the adaptive action of moderate repeated stress on the HPA-axis response, unbiased estimation of increased stress levels in animals during the pre-slaughter period is difficult. Also for metabolic markers of stress such as lactate, short-term versus longer-term stress resulted in differential responses in slaughter pigs^11,12^. In humans, it has been shown that parameters of the somatotropic axis are appropriate for the characterization of different stages of disorders or impairment of health^13,14^. Growth hormone (GH) is a primary regulator of vertebrate growth and metabolism. In mammals, it is assumed that acute physical stress, energy restriction, or acute phase of severe illness induce an amplification of GH secretion and increased levels of GH^13,15,16^. Although GH response to psychological stress is rarely seen^17^, studies in humans indicate that GH response is positively correlated with higher post-stress levels of anxiety^18^. In addition to direct effects, GH also affects body growth and metabolism indirectly through the stimulation of insulin-like growth factor (IGF) production in a number of tissues including the liver^19^. In blood, IGF is bound to IGF-binding proteins (IGFBPs) which control IGF availability, but also have IGF-independent functions^20^. As IGFBPs are sensitive markers to detect changes of the GH-dependent growth^21,22^, they are important biomarkers for diagnostics and treatment studies in humans^14^. In addition to the central role of the IGF-system in linking nutritional intake with somatic growth^23–25^ it is known that glucocorticoids influence levels of IGF-1 and IGFBPs^26–28^ suggesting an interference of acute stress with the IGF system. However, to our knowledge, stress-induced changes of the IGF-system have never been studied comprehensively in pigs yet.

Therefore, the aim of the study was to assess the effects of transport stress factors on the plasma circulating levels of IGF-1, IGF-2, and IGFBPs in pigs during different stages of commercial pig transportation in Germany. In addition, the effect of repeated stress by snaring three times within the pre-slaughter period of 28 h was investigated providing an experimental model for repeated acute stress^8^ in the pre-slaughter period. IGFBPs were evaluated using quantitative Western ligand blotting (qWLB) which provides complex information on IGFBP-profiles in blood plasma^29^. Furthermore, the calculation of distinct ratios, as IGFBP-3/-2 ratio or IGF-1/total amount of IGFBPs, provides additional information about regulation of the somatotropic axis and IGF-1 bioavailability, respectively. To validate the quality of results related to the activation of the IGF-system in response to stress, a set of established serum markers and stress vocalization reflecting the acute physiological stress response, inflammatory pathways and energy metabolism were also evaluated.

## Results

### Effects of sampling time on IGF-system, stress response, and energy metabolism

**Figure 1:**
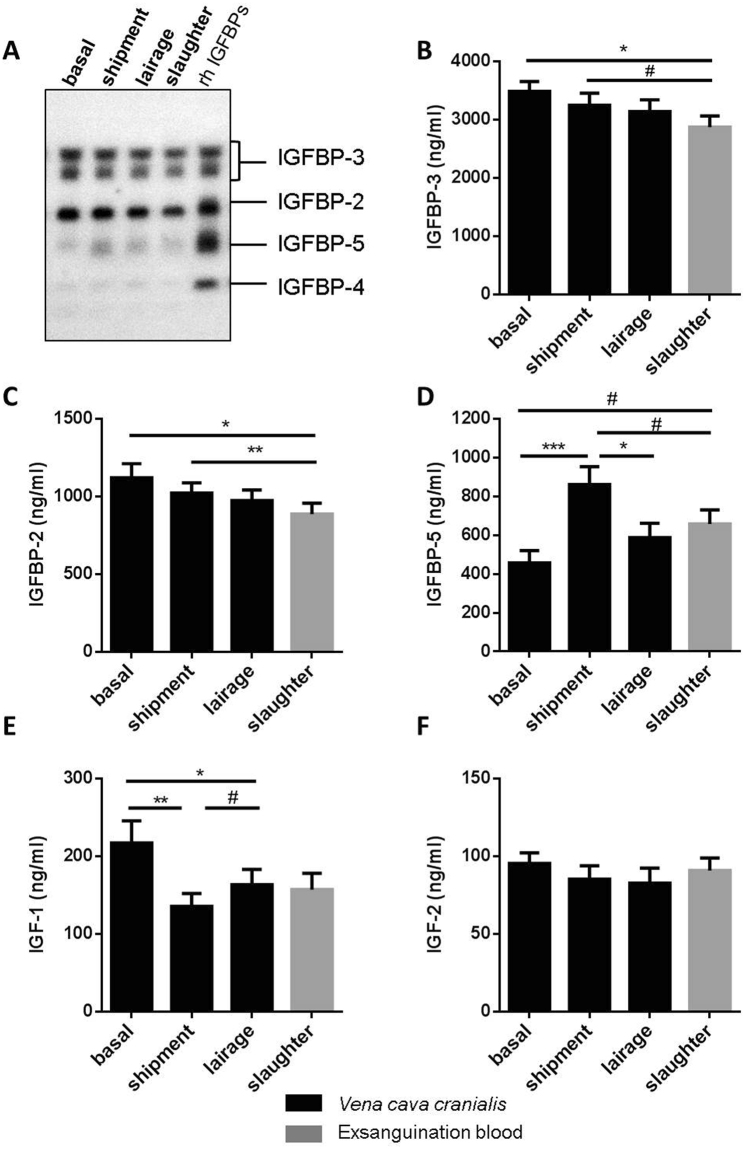
IGF-system at different sampling times of transportation procedure: IGFBP-profile (**A**), IGFBP-3 ((**B**); n = 31), IGFBP-2 ((**C**); n = 31), IGFBP-5 ((**D**); n = 31), IGF-1 ((**E**); n = 13) and IGF-2 ((**F**); n = 13). Quantitative data are presented as LS-means+ SE. rh: recombinant human. *p < 0.05, **p < 0.01, ***p < 0.001, ^#^p < 0.1.

**Figure 2:**
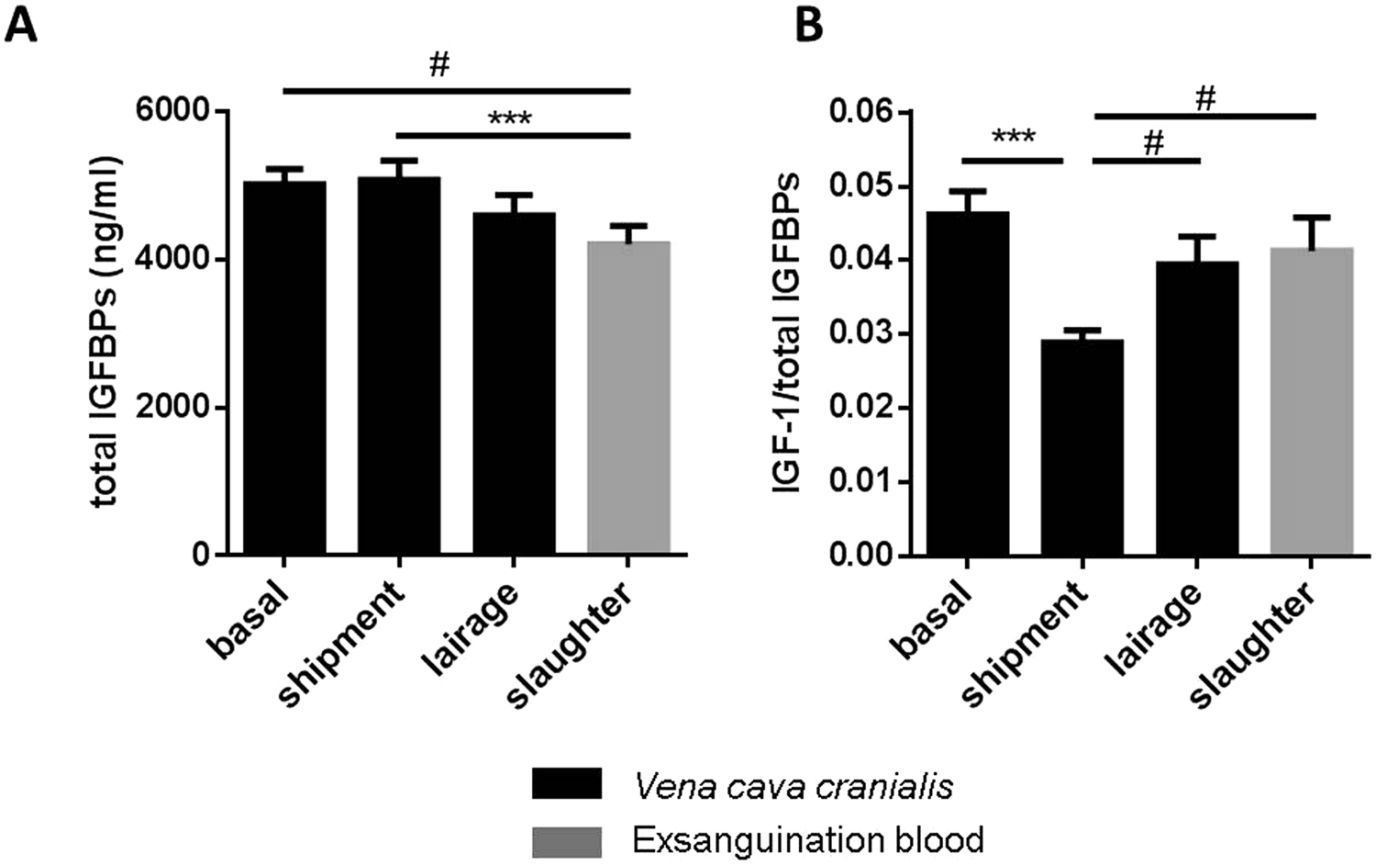
The total amount of IGFBPs as marker for IGF-binding capacity ((**A**); n = 31) and the ratio of IGF-1/total IGFBPs as indicator for IGF-1 bioavailability ((**B**), n = 13) during different stations of transport. Data are presented as LS-means+ SE. *p < 0.05, **p < 0.01, ***p < 0.001.

**Figure 3:**
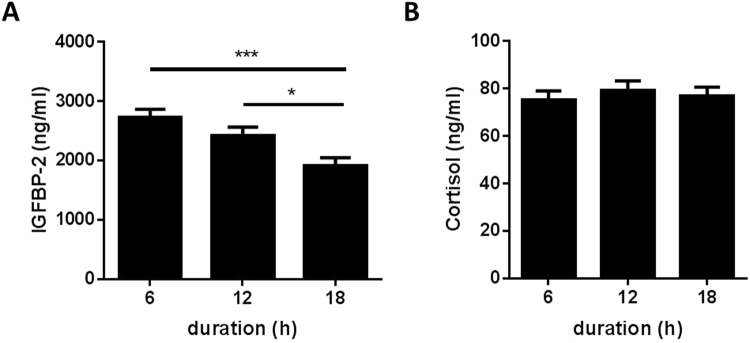
Effect of transport duration on plasma IGFBP-2 ((A); n = 240) and cortisol ((**B**); n = 90). IGFBP-2 was analyzed using quantitative Western ligand blot. Cortisol was analyzed using LC-MS/MS. Data are presented as LS-means+ SE. *p < 0.5, **p < 0.01, ***p < 0.001.

**Figure 4:**
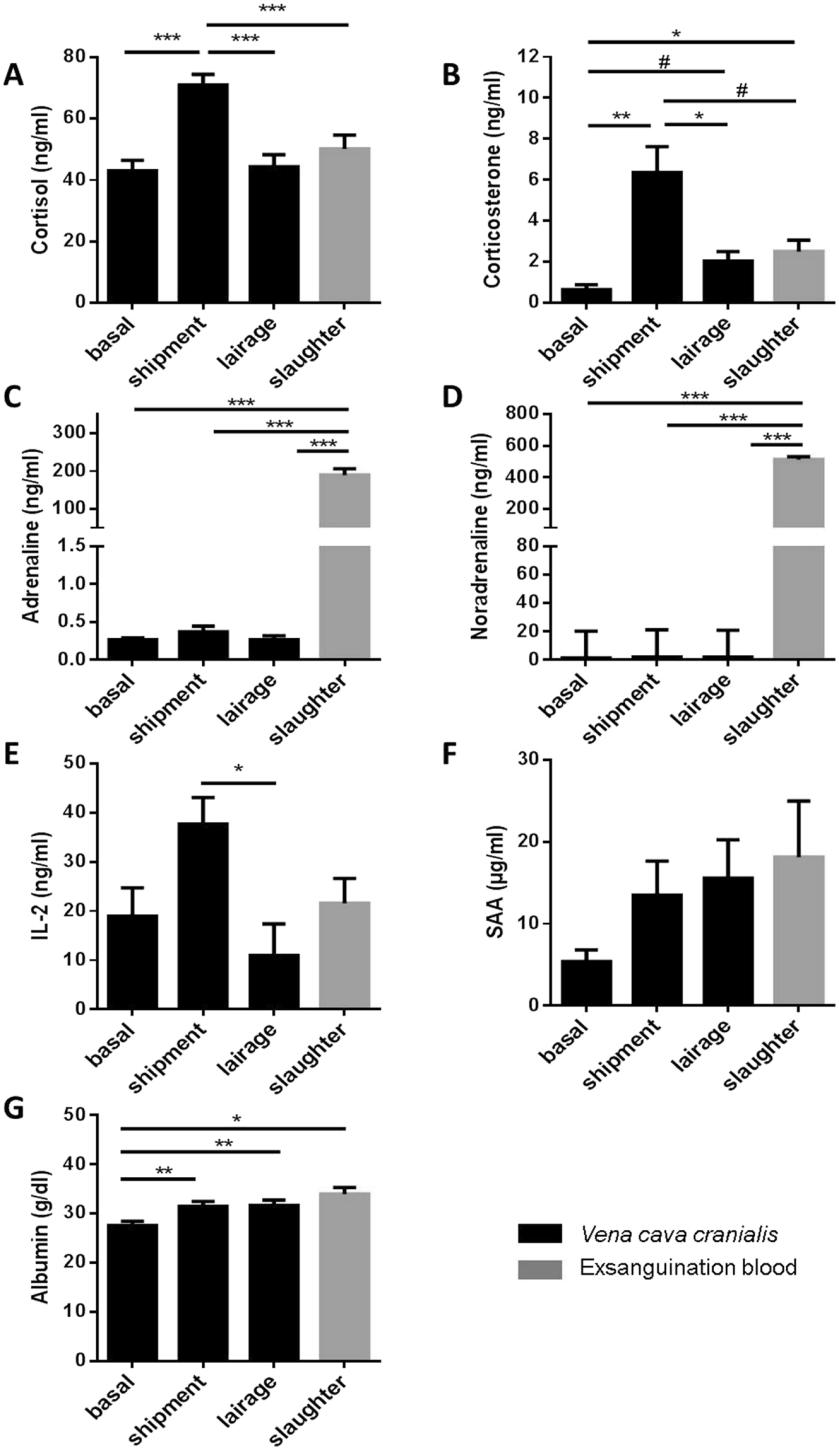
Stress and inflammation associated parameters at different sampling times of commercial transportation procedure: Plasma concentrations of cortisol (**A**), corticosterone (**B**), adrenaline (**C**) and noradrenaline (**D**), IL-2 (**E**), SAA (**F**) and albumin (**G**). Cortisol, IL-2 and SAA were analyzed with ELISA. Corticosterone was analyzed using LC-MS/MS. Adrenaline and noradrenaline were analyzed using HPLC. Albumin was analyzed using an enzymatic spectrophotometric assay. All Data are presented as LS-means+ SE. Cortisol, adrenaline, noradrenaline, SAA: n = 31 per sampling time. Corticosterone, IL-2, albumin: n = 13 per sampling time. ^#^p < 0.1, *p < 0.05, ** p < 0.01, ***p < 0.001.

**Figure 5:**
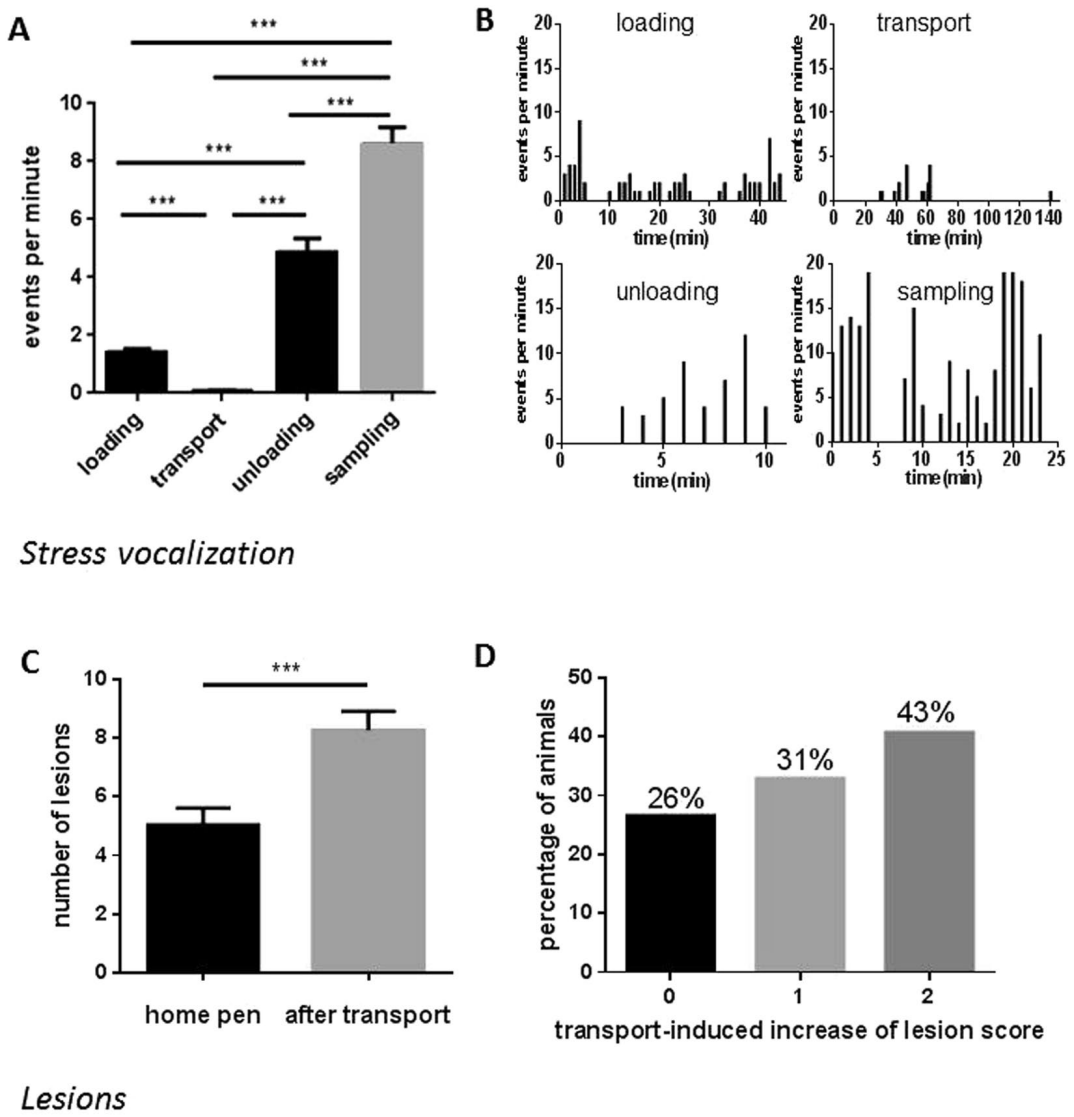
Stress vocalization and evaluation of lesions at different events of pig transport. (**A**) Stress vocalization, presented as LS-means + SE of every event, respectively. (**B**) Exemplary data of stress vocalization during different events of trial 2. (**C**) The number of lesions measured in home pen and after transport, presented as LS-means + SE. (**D**) Percentage of animals per category of transport-induced increase of lesion score. The stress vocalization was analysed using STREMODO and includes all animals in the range of sound recorder. Lesions: n = 65. *p < 0.05, **p < 0.01, ***p < 0.001.

In non-transported pigs on the farm, food removal for a period of 19 h did not affect plasma concentrations of IGF-1, IGFBP-2, and IGFBP-3 (Suppl. Figure 1A). In addition, the time of day between 8 a.m. and 2 p.m. had no effect on IGF-1 or IGFBP-2 in non-transported pigs (Suppl. Figure 1B). However, at 12 p.m. reduced concentrations of IGFBP-3 were present in non-transported pigs if compared to 8 a.m. (p < 0.01). The concentrations of IGFBP-2 and IGFBP-3, which appear to be the dominant IGFBPs in porcine plasma (Fig. 1A), decreased in the course of the pre-slaughter period between 9.45 a.m. and 1.15 pm while the pigs rested in the lairage of slaughterhouse. The lowest levels of both IGFBP-2 and -3 were measured in slaughter blood. More specifically, plasma IGFBP-3 levels were lower at slaughter when compared with basal levels (p = 0.023, Fig. 1B), while IGFBP-2 concentrations were decreased after slaughter compared to basal levels (p = 0.004) and levels after shipment (p = 0.025, Fig. 1C). By contrast, IGFBP-5 was increased after shipment compared to basal levels (p = 0.0004) followed by a decrease after lairage (p = 0.010, Fig. 1D). In plasma, IGF-1 was reduced after shipment (p = 0.001) and after lairage (p = 0.035) compared to basal concentrations measured in the home pen (Fig. 1E), whereas IGF-2 was not affected by the sampling time (Fig. 1F). The total amount of all plasma IGFBPs (IGFBP-2, -3 and -5), simultaneously quantified by qWLB, was reduced in exsanguination blood after CO_2_ stunning, compared to levels after shipment (p = 0.0006, Fig. 2A). In contrast, the ratio of IGF-1/total IGFBPs just as IGF-1/IGFBP-3 were decreased exclusively after shipment (p = 0.0008, p = 0.008, respectively) compared to basal levels in the home pen on the farm (Fig. 2B). The ratio of IGF-1/IGFBP-5 decreased after shipment (p = 0.002) and after slaughter (p = 0.034) compared to basal concentrations (Suppl. Table 1). The sampling time had no effect on the ratio of IGF-1/IGFBP-2 and IGFBP-3/IGFBP-2. We next asked, if IGFBP-2 may indicate different levels of stress and tested different lengths of transport duration in slaughter pigs (Fig. 3). After 18 h of shipment, the IGFBP-2 concentrations in plasma were significantly reduced if compared to pigs transported for 6 or 12 h, whereas cortisol was similar in all groups irrespective of transportation time. Cortisol was increased after shipment compared to basal levels (p = 7.4E-06), lairage (p = 3.3E-05) and slaughter (p = 0.003, Fig. 4A). Corticosterone (Fig. 4B) was increased after shipment (p = 0.004) followed by a decrease (p = 0.043) at lairage. Distinct to cortisol, corticosterone was not normalized after lairage and was still elevated after slaughter (p = 0.049) compared to basal levels. Adrenaline and noradrenaline (Fig. 4C,D) were massively increased in exsanguination blood (p < 1.2E-12). IL-2 (Fig. 4E) was reduced after lairage if compared to shipment (p = 0.033). The concentrations of SAA were statistically not different between the sampling times (Fig. 4F). Albumin (Fig. 4G) also increased with prolonged sampling time compared to basal blood levels (p = 0.0019, p = 0.006, p = 0.013). Glucose, cholesterol, triglycerides, and lactate (Suppl. Figure 2A–D), were not significantly different in the pre-slaughter period but were increased in exsanguination blood compared to basal levels (p = 0.0008, p = 0.076, p = 0.005, p = 2.35E-05). Stress vocalization was significantly different during loading, transport, unloading, and blood sampling (Fig. 5A,B). The highest and lowest levels of vocalization were recorded during sampling and shipment, respectively. The number of lesions per pig increased after transport compared to home pen (p = 7.56E-11). A percentage of 26% of the gilts had no transport-induced increased lesion score whereas 31% of animals had an increase of score 1 and 43% of score 2 (Fig. 5C,D).

### Effects of repeated blood sampling by snaring on plasma parameters, meat and carcass quality in exsanguination blood

Slaughter blood was compared between pigs treated with repeated blood sampling using snaring (treatment group, n = 31) and pigs without blood sampling in the pre-slaughter period (control group, n = 34).The pigs of the treatment group were snared three times before slaughtering, in the home pen on farm (9:00 a.m.), after unloading (9:45 a.m.) and after lairage in the slaughterhouse (11:45 a.m.). Repeated blood sampling affected parameters of IGF-system and stress response. LS-means and p-values of pairwise comparison of plasma parameters, carcass and meat quality between the treatment group (with repeated blood sampling before slaughter) and controls (no repeated blood sampling before slaughter) are presented in Table 1. IGFBP-3, IGF-1 and the IGFBP-3/IGFBP-2 ratio and were higher in animals with repeated blood sampling compared to controls. Corticosterone and lactate were reduced whereas noradrenaline was elevated in the treatment group compared to control. Carcass and meat quality were not significantly affected by the repeated blood sampling.

**Table 1:** Effect of repeated blood sampling (three times) in the pre-slaughter period on plasma parameters and meat quality in exsanguination blood, collected directly after slaughter.

Parameters	Treatment group	n	Control group	n	p-value
**Plasma**					
IGFBP-3 (ng/ml)	2855.21 ± 165.38	31	2362.57 ± 152.13	34	0.0323
IGFBP-2 (ng/ml)	904.46 ± 57.96	31	998.26 ± 53.31	34	0.2384
IGFBP-5 (ng/ml)	653.20 ± 86.31	31	489.71 ± 80.41	34	0.1723
IGF-1 (ng/ml)	157.44 ± 17.31	13	108.07 ± 15.74	14	0.0476
IGF-2 (ng/ml)	82.82 ± 8.63	13	82.60 ± 7.66	14	0.9845
Total IGFBPs (ng/ml)	4098.24 ± 232.44	31	3725.65 ± 289.55	34	0.2180
IGF-1/total IGFBPs	0.04 ± 0.004	13	0.03 ± 0.004	14	0.0616
IGF-1/IGFBP-3	0.06 ± 0.01	13	0.05 ± 0.01	14	0.1301
IGF-1/IGFBP-2	0.19 ± 0.02	13	0.13 ± 0.02	14	0.0519
IGF-1/IGFBP-5	0.21 ± 0.05	13	0.16 ± 0.03	14	0.3608
IGFBP-3/IGFBP-2	3.44 ± 0.29	31	2.60 ± 0.27	34	0.0392
Glucose (mg/dl)	157.11 ± 10.51	13	153 ± 9.54	14	0.8054
Albumin (g/dl)	33.96 ± 1.21	13	33.07 ± 1.10	14	0.5873
IL-2 (ng/ml)	21.52 ± 4.98	13	30.73 ± 5.65	14	0.2435
Cortisol (ng/ml)	50.49 ± 5.03	31	50.73 ± 4.67	34	0.9720
Corticosterone (ng/ml)	2.51 ± 0.60	13	4.47 ± 0.57	14	0.0273
Lactate (mmol/l)	5.90 ± 0.66	31	8.57 ± 0.61	34	0.0043
Cholesterol (g/l)	1.26 ± 0.05	13	1.26 ± 0.04	14	0.9240
Triglycerides (g/l)	0.57 ± 0.05	13	0.45 ± 0.05	14	0.0819
Serum Amyloid A (µg/ml)	17.42 ± 4.03	31	7.60 ± 4.78	34	0.1694
Adrenaline (ng/ml)	189.48 ± 14.70	31	163.26 ± 13.52	34	0.1945
Noradrenaline (ng/ml)	511.62 ± 33.82	31	409.48 ± 31.11	34	0.0301
**Carcass quality**					
Hot carcass weight (kg)	93.05 ± 1.16	31	92.93 ± 1.08	34	0.9352
Lean content (kg)	57.86 ± 0.48	31	58.50 ± 0.46	34	0.3388
Lean yield (%)	62.49 ± 0.93	31	63.29 ± 0.89	34	0.5346
**Meat quality**					
pH1	6.42 ± 0.03	31	6.50 ± 0.03	34	0.0713
pH2	5.48 ± 0.03	31	5.49 ± 0.03	34	0.7362
Drip loss (%)	2.30 ± 0.14	31	2.40 ± 0.13	34	0.6061

Data are presented as LS-means ± SE and p-value is given for the pairwise comparison between the treatment group (with repeated blood sampling by snaring) and the control group (without repeated blood sampling) using Tukey-Kramer procedure.

### Correlation of IGF-compounds and established markers of stress and metabolism

With only one exception, exclusively negative correlations were found between compounds from the IGF-system and established biomarkers of stress and metabolism (Table 2). Negative correlations were existent between IGF-1, IGF-2, IGFBP-2 or IGFBP-3 on one hand and cortisol, corticosterone, glucose, albumin, lactate, SAA, cholesterol or triglycerides on the other (p < 0.05). As the only exception, IGFBP-5 was positively regulated with cortisol (p < 0.001). Cortisol correlated positively with albumin and lactate in all samples (P < 0.05).

**Table 2:** Rank correlation of metabolic and stress related parameters after Spearman.

Parameter 1	Parameter 2	Sample size	p-value	R_Spearman_	Correlation
IGFBP-3	IGFBP-2	156	<0.001	0.40	moderate
IGFBP-3	total IGFBPs	156	<0.001	0.94	high
IGFBP-3	IGF-1	57	<0.001	0.45	moderate
IGFBP-3	Glucose	67	<0.001	−0.48	moderate
IGFBP-3	Lactate	156	<0.01	−0.24	moderate
IGFBP-3	IGFBP-3/-2 ratio	156	<0.001	0.56	distinct
IGFBP-2	total IGFBPs	156	<0.001	0.55	distinct
IGFBP-2	SAA	156	<0.05	−0.16	low
IGFBP-2	IGFBP-3/-2 ratio	156	<0.001	−0.46	moderate
IGFBP-5	total IGFBPs	130	<0.001	0.32	moderate
IGFBP-5	Cholesterol	58	<0.05	−0.33	moderate
IGFBP-5	Cortisol	130	<0.001	0.31	moderate
total IGFBP	TG	65	<0.01	−0.37	moderate
total IGFBP	Glucose	67	<0.001	−0.44	moderate
total IGFBP	Lactate	156	<0.01	−0.22	moderate
total IGFBP	IGFBP-3/-2 ratio	156	<0.001	0.36	moderate
IGF-1	total IGFBPs	57	<0.01	0.41	moderate
IGF-1	Cortisol	57	<0.001	−0.31	moderate
IGF-1	Corticosterone	57	<0.05	−0.32	moderate
IGF-1	IGF-2	57	<0.001	0.38	moderate
IGF-1	Glucose	57	<0.001	−0.45	moderate
IGF-1	IGFBP-3/-2 ratio	57	<0.001	0.49	moderate
IGF-1	Lactate	57	<0.001	−0.43	moderate
IGF-1	Albumin	57	<0.05	−0.31	moderate
IGF-2	Lactate	57	<0.05	−0.27	moderate
Albumin	Cortisol	57	<0.05	0.29	moderate
Albumin	Corticosterone	57	<0.001	0.57	distinct
Albumin	Lactate	66	<0.05	0.31	moderate
Albumin	IGFBP-3/-2 ratio	66	<0.05	−0.28	moderate
Glucose	TG	65	<0.001	0.60	distinct
Glucose	Lactate	67	<0.001	0.59	distinct
Glucose	IGFBP-3/-2 ratio	67	<0.001	−0.47	moderate
Cortisol	Corticosterone	65	<0.001	0.67	distinct
Cortisol	Lactate	155	<0.01	0.25	moderate
Corticosterone	IGFBP-3/-2 ratio	65	<0.01	−0.32	moderate
Lactate	IGFBP-3/-2 ratio	156	<0.01	−0.21	moderate

Only correlations with a p-value < 0.05 are presented. The Spearman correlation coefficient (R_Spearman_) was classified as low (0,0 ≤ R ≤ 0,2), moderate (0,2 < R ≤ 0,5), distinct (0,5 < R ≤ 0,8) and high (0,8 < R ≤ 1,0).

## Discussion

The identification and application of novel biomarkers are needed to ensure reliable monitoring of animal welfare. In the present study, we have assessed the effects of shipment on compounds from the IGF-system. According to the hypothesis of Mesotten and van den Berghe^14^, the IGF-system and, in particular the signature of different IGFBPs, provide significant information in relation to disturbances of homeostasis in animals.

### Effect of shipment

Similarly to previous works^6,30^, in this study, plasma concentrations of cortisol and corticosterone increased after transportation, including loading and unloading, which may be considered as a response of the HPA-axis to transport associated stress. In addition, increased concentrations of IL-2 have been found in plasma collected after unloading suggesting an effect of transportation on the pigs’ immune function as already reported^31,32^. With respect to stress vocalization, the data of this study suggest that the loading and unloading procedures are more stressful than road driving, which also is in agreement with previous reports^6^. Similar to humans, cortisol also in pigs was the predominant circulating glucocorticoid whereas corticosterone was found at more than 20-fold lower concentrations^33^. In mammals, glucocorticoids are thought to reduce circulating IGF-1^26^. Interestingly, reduced serum concentrations of IGF-1 and IGFBP-5 were observed after 2 h of shipment. In post-weaning piglets at an age of 20 days after birth, 3h-shipment had no effect on serum IGF-1 concentrations^34^. By contrast in sows, heat stress over 57 days reduced serum concentrations of IGF-1 if compared to shorter exposure times of heat stress, although the IGFBPs have not been assessed in that study^35^. Also, acute stress reduced serum IGF-I concentrations in adult Yorkshire pigs^36^. In this study, 5 min of restraint stress depressed serum IGF-1 concentrations for about 20% 150 min after snaring. According to our knowledge, IGFBPs have not been assessed under conditions of stress in pigs. However, in fish, both stress and the administration of dietary cortisol decreased levels of plasma IGF-1 after 2 h but increased plasma levels of up to four different IGFBPs, which were not specified in these studies^27,28^. These findings support the biomarker potential of IGF-system for evaluation of stress in vertebrates. In any case both, higher levels of plasma IGFBP-5 and lower levels of plasma IGF-1 after shipment, may be related to increased cortisol and corticosterone plasma levels also shortly after transport.

The concentrations of other IGFBPs were not affected by shipment over a period of 2 h. However, in a separate experiment with longer transportation, IGFBP-2 concentrations in plasma from pigs were reduced over time, whereas cortisol was similar after 6, 12, and 18 h of transport. IGFBP-2 is regulated by hormones (GH, steroids, insulin, leptin) but also by proteolysis^37^. Accordingly, these parameters may be considered as effectors of IGFBP-2 concentrations under conditions of elevated stress. All animals were fasted to a similar extent for 20–24 hours and plasma was derived from slaughter blood at the same time in the morning. Thus, IGFBP-2 concentrations may decrease over time due to the length of shipment, whereas cortisol did not change with prolonged duration of transportation. Thus, we have reason to assume dose-response of the plasma IGFBP-2 concentrations in response to stress. By contrast, cortisol levels possibly due to habituation have no biomarker value under conditions of prolonged stress in transported slaughter pigs.

### Effects of lairage and slaughter

During lairage, serum concentrations of corticoids and IL-2 normalized indicating efficient recovery after shipment. In addition, also IGF-I and IGFBP-5 returned to normal if compared to basal levels before transport, indicating responsiveness of IGF-I and IGFBP-5 to intermediate stress. All metabolic parameters were unaffected by treatments other than slaughter. The massive increase of catecholamines in slaughter blood is due to CO_2_ stunning. The significant reduction of IGFBP-2 in slaughter blood is considered being more the consequence of the prolonged stressful procedure and less due to the acute condition of stress in the CO_2_ stunner. This assumption is supported by the perpetual decrease of plasma IGFBP-2 concentrations during prolonged transport duration from 6 h up to 18 h of shipment.

IGFBP-2 was reduced also in slaughter blood if compared to basal levels. Because food removal over a period of 19 h did not affect IGFBP concentrations in a control experiment performed with non-transported pigs, these results cannot be explained by the effects of dietary energy levels and fasting status. By contrast, prolonged fasting is well known to regulate IGFBP-2^37^. Accordingly, in newborn pigs prolonged fasting for 48 h decreased IGFBP-2 concentrations measured by Western ligand blotting^24^. Fasting for 70 h also reduced serum concentrations of IGF-1 and IGFBP-3 but increased serum levels of IGFBP-2 in sheep^38^. Besides IGFBP-2 also IGFBP-3 was decreased in slaughter blood. However, this reduction also may be due to circadian effects, because at 12:00 o’clock lower IGFBP-3 concentrations have been found if compared to IGFBP-3 levels in the morning at 8:00 a.m.

Skin damage is associated with higher pH values and darker meat colour (potentially dark, firm, dry; DFD) resulting from increased glycogen depletion in the muscle^39^. In the current study, lesion scoring revealed only a slight increase of skin damage after transportation. Furthermore, pH1, pH2, and drip loss analyses revealed no stress induced effect on meat quality^40,41^ which is not surprising considering the high standardized pre-slaughter conditions with no mixing in lairage and handling of pigs in small groups without electric prodding and push gates in lairage^3,39^ applied in this study. In the present study, CO_2_-stunning induced a significant increase of plasma glucose as a result of the high release of catecholamines such as adrenaline^42^. The stress response during slaughtering is influenced by breed-specific traits, pre-slaughter conditions such as handling or lairage time, and the type of stunning system^43,44^, which have to be considered for the evaluation of stress-associated biomarkers.

### The effect of restraint stress

Short-term restraint stress by snaring and *vena cava* blood sampling increased concentrations of catecholamines, cortisol, lactate, and glucose in pigs^45–47^. Therefore, we asked whether repeated restraining for blood sampling of the treatment group influenced blood parameters and meat quality after slaughtering as an experimental model for repeated short-term stress. The results indicate that repeated acute stress by snaring attenuates the corticosterone response to acute slaughter stress. In rats, it was described that repeated short-term stress by restraint potently attenuates acute stress- induced activation of the HPA axis, indicating an adaptive effect of moderate stress on the HPA axis response to acute stress^10^. This attenuation might be important regarding the validity of glucocorticoids as biomarkers for pre-slaughter stress of pigs in exsanguination blood. In contrast to glucocorticoids, in the present study, noradrenaline was increased in repeatedly restrained pigs suggesting an enhanced stimulation of sympathetic-adrenal medullary system by slaughter-induced stress. In rats, it is described that exposing repeatedly stressed animals to an unrelated stressor results in an enhanced sympathetic-adrenal medullary response indicating a sensitization of this axis^48,49^. Both habituation and sensitization are described as adaptive processes that allow the organism to physiologically cope with prolonged or intermittent stress exposure preventing deleterious actions but maintaining response flexibility to new threats^50^. In the current study, IGF-1, IGFBP-3 and the ratio of IGFBP-3/-2 were higher in exsanguination blood of repeatedly restrained pigs indicating a modulation of IGF-system by repeated stress. It is described, that glucocorticoids regulate IGF and IGFBP-expression in a direct or indirect manner^51^. Glucocorticoids, such as cortisol or dexamethasone, are shown to down regulate IGF-I and IGFBP-3 levels aiming at inhibiting IGF anabolic activity^51^. The higher levels of corticosterone, found in the exsanguination blood of pigs not subjected to repeated blood sampling may explain their lower levels of IGF-1 and IGFBP-3 at slaughter in this study. As there was no effect of repeated blood sampling on plasma IGFBP-2 concentrations, the increased levels of IGFBP-3 led to an increased IGFBP-3/IGFBP-2 ratio, which is considered as a sensitive marker for GH-induced somatic growth and metabolic homeostasis in humans. During long-term critical illness or low physical performance this ratio decreases^14,52^ due to a suppression of GH release^13,16^. Because of the complex regulation of IGF-system, short term effects of acute and repeated stressors on IGFBP-3/IGFBP-2 ratio has still to be investigated. However our study clearly demonstrates acute regulation of the IGF-system during animal transport and the significant correlations with established biomarkers of stress also support novel biomarker potential of the IGF-system.

To summarize and conclude, the signature of the IGF-system contains a complex set of information for specific segments in the transport process from farm to slaughter (Table 3). In both transportation studies, plasma concentrations of IGFBP-2 were reduced over time suggesting dose-dependency of this parameter. Beyond other IGFBPs, IGFBP-5 concentrations were highly dynamic between selected segments of the transportation chain and similar to alterations of IL-2 and glucocorticoids. Finally, plasma IGF-1 concentrations are flexible during animal shipment and may increase or decrease during transportation, while IGF-2 was not regulated acutely during pig transportation. Our results provide substantial evidence that compounds from the IGF-system are specifically regulated by different stressors in the transportation chain. In particular, our results provide evidence that glucocorticoids are less informative under conditions of repeated or prolonged stress. Compounds from the IGF-system may thus represent physiologically relevant biomarkers of repeated or prolonged stress e.g. with extended periods of shipment. Monitoring compounds from the IGF-system in pigs may generate novel biomarker information and improve current standards of animal husbandry. Due to major effects on economical traits, consideration of IGF-compounds in farm animals may guide development of animal husbandry in order to integrate both ethical and economic issues in the future.

**Table 3:** Conditional biomarker potential of physiological parameters.

Factor	Effect on physiological parameters/biomarker potential
Short-term fasting	no effect on IGF-compounds assessed
Time of the day	IGFBP-3 ↓
Shipment versus basal	Corticoids ↑
	IGF-1 ↓, IGFBP-5 ↑
Lairage versus shipment	Corticoids ↓, IL-2 ↓
	IGFBP-5 ↓
Slaughter versus lairage	Catecholamines ↑, Glucose ↑, Triglycerides ↑, Lactate ﻿↑﻿, Cholesterol ↑
Slaughter versus basal	Catecholamines ↑, Glucose ↑, Lactate ↑, Triglycerides ↑
	IGFBP-2↓
Restraint stress	Corticosterone ↓, Lactate ↓,
	IGF-I ↑, IGFBP-3 ↑, IGFBP-3/IGFBP-2 ↑
Transport duration	IGFBP-2↓

## Materials and Methods

### Animals and pre-slaughter conditions

All experimental procedures adhered to the current guidelines and were approved by the Landesamt für Landwirtschaft, Lebensmittelsicherheit und Fischerei Mecklenburg-Vorpommern as the responsible authorities (reference number LALLF M-V/TSD/7221.3-2-033/14). The pigs were fed a commercial diet (12.8 MJ ME/kg, 15% crude protein, CP) and had free access to water. The animals were grouped at least 4 months before shipment. Social group structure was maintained during the whole experimental procedure on farm, truck and slaughterhouse. A total of 65 market weight (115 ± 7.9 kg) crossbred gilts (Danish Landrace x Yorkshire sows x Piétrain boars, homozygous *NN* for *Hal* gene = stress resistant) were randomly selected at a commercial finishing farm and shipped (2 h) to a commercial slaughterhouse (VION, Perleberg, Germany). The ambient temperature during shipment was −5–10 °C. At the farm the pigs were withdrawn of feed for 18 h (since 12.00 pm day 1) and loaded to a commercial truck by a trained crew using boards. The upper decks of the truck were lift up hydraulic. On arrival at the slaughterhouse, pigs were unloaded through a plane ramp using boards. Pigs were kept in lairage for 3 h during which they had free access to water and water sprinkling. For blood sampling pigs were separated in 2 pens (2.5 m^2^/pig) and control pigs were separated in a third pen (1.25 m^2^/pig). At the end of lairage, groups of 5 pigs were driven with boards to a mechanical driveway forward to the CO_2_ stunner (Butina CO_2_ backloader system, Holbaek, Denmark, 92% CO2) and subsequently exsanguinated in a vertical position. An overview about the experimental design is given in Suppl. Figure 3. In order to test the effect of transport duration on plasma concentrations of IGFBP-2 and cortisol, we analysed samples of an independent shipment study in slaughter pigs^53^. All pigs (total n = 240) had no access to food for 20–24 h and arrived at the slaughter plant the same time as described before^53,54^. The effects of food removal and time of day on the IGF-system were assessed in non-transported pigs as described before^55^. Thereby, blood sampling was performed in catheterized pigs every two hours starting 8 a.m. until 8 a.m. next morning.

### Blood collection

Exsanguination blood was gathered from all pigs included in the present study. Basal blood samples from *vena cava cranialis* were collected in the home pen (21 h before transport). Therefore the pigs were snared and the neck was stretched well upwards for punction of the vena. A total of 31 pigs (treatment group) were used for repeated blood sampling collected in the home pen (basal), directly after unloading (shipment) as well as after lairage (lairage). Repeated bleeding was not performed in a second group of 34 pigs (control group) in order to study the potential effect of the bleeding procedure. Blood samples (approximately 8 ml) were collected in ice-cooled tubes containing 200 µl of 0.1 M EDTA and centrifuged at 2,000 g for 10 min at 4 °C. Then, the plasma was stored in dry ice for transportation to laboratory unit and stored at −20 °C until analysis.

### Quantitative Western ligand blotting

IGFBP-2, -3 and 5 were analyzed by quantitative Western ligand blot (qWLB) analysis as described previously^29^. Due to low abundance, IGFBP-4 was detected but not quantified in the porcine plasma. The analytical range for each IGFBP was 150–15000 ng/ml. Inter-and intra-assay coefficients of variation (CV) were determined by measuring artificial serum samples spiked with low (500 ng/ml) and high (3000 ng/ml) concentrations of IGFBPs. The intra-assay CV (n = 9) for IGFBP-2,-3 and -5 was <15% at high and low concentrations. The inter-assay CV (n = 8) for all IGFBPs was <20% at low and <15% at high concentrations according to recommendations of EMA guideline^56^. In order to account for the higher variances of Western blotting data compared to ELISA data we decided to assess all samples by quantitative Western ligand blotting.

### ELISA assays

All ELISA assays were performed using commercially available ELISA Kits according to the manufacturer’s instructions. Concentrations of IGF-1 and IGF-2 were analyzed in the plasma samples using ELISA Kits E20 and E30 (Mediagnost, Reutlingen, Germany). Cortisol was determined with EIA 1887 (DRG Instruments GmbH, Marburg, Germany) and cross validated with LC-MS/MS revealing a correlation coefficient of r = 0.926 (Spearman correlation). Plasma concentrations of IL-2 were analyzed by Bioglobe GmbH (Hamburg, Germany) using the Bio-Plex Pro™ Human Cytokine Assay (Biorad, Hercules, U.S.A.). Serum amyloid A (SAA) was determined using a Phase SAA ELISA kit (Tridelta Development Ltd., Maynooth, Ireland).

### LC-MS/MS and HPLC

Plasma concentration of corticosterone was analyzed using LC-MS/MS technique described previously^57^. The intra-assay CV at different concentrations (5 ng/ml, 50 ng/ml, 500 ng/ml) for corticosterone were 13.05%, 10.98% and 4.64%, respectively. The inter-assay CV for 100 ng/ml (n = 20) was 5.57%. Plasma concentrations of adrenaline and noradrenaline were analyzed using HPLC with electrochemical detection after extraction from plasma by absorption on aluminum oxide^58^. The intra- and inter-assay CV were 3.1% and 5.5%, respectively, for adrenaline and 2.0% and 2.5%, respectively, for noradrenaline.

### Enzymatic spectro-photometric assays

Plasma lactate was determined by an enzymatic-spectrophotometric assay (Labor + Technik Eberhard Lehmann GmbH, Berlin, Germany). Triglycerides (TG), cholesterol, glucose and albumin were assayed in plasma samples via enzymatic coloured test by using commercial kits (triglycerides: No. LT-TR 0015, total cholesterol: No. LT-CH 0031, glucose: LT-GL 0251; albumin: LT-AB 0103; Labor & Technik Eberhard Lehmann Berlin, Germany, respectively) as described^59^.

### Stress vocalization

Stress vocalization was recorded as described before^60^. In brief, a commercially available voice recorder was installed at the loading ramp of the truck during loading and unloading of the animals and near the waiting pen during blood sampling. A second voice recorder was fixed within the animal truck which was a self-developed voice recorder for harsh environment based on a digital signal processing (DSP) board. The recordings were divided into the sections loading, unloading, transportation and sampling. The WAV-files of the recordings were retrospectively analysed using the STREMODO software. The software uses a neuronal network to classify time windows of 50 ms into stressful vocalization and non-stressful vocalisation. Stress detections in successive windows were taken as a single event, while allowing interruptions of less than 200 ms. The STREMODO software can give false positive classifications for high-pitched metallic or air flow sounds. Such sounds occur most likely within the truck and during transportation. They were not eliminated manually. However, very short detection events with durations of less than 150 ms were excluded from the statistical evaluation.

### Evaluation of carcass and meat quality

Skin lesions on the body of all pigs were registered by the same observer in the home pen before transport and after the second blood sampling essentially as described^61^. The total number of lesions was evaluated in home pen and after transport. The increase of lesions, that were not present before transport, were counted and lesion scores assigned as follows: 0 – no skin injuries, 1 – one to three wounds; 2 – four or more injuries. The hot carcass weight and lean body content was standardized assessed in the slaughterhouse. The pH value was evaluated in the *musculus longissimus dorsi* by assessing the pH value of all pigs after 45 min (pH1) and 24 h (pH24) post-mortem. Drip loss was analysed in *musculus longissimus dorsi* according to standard procedures^62^.

### Statistical analysis

Statistical analyses were performed using SAS software version 9.3 (SAS, Cary, NC, USA). Descriptive statistics and tests for normality were calculated with the UNIVARIATE procedure. The data of all blood parameters were evaluated by ANOVA using MIXED procedure. Repeated measures on the same animal were taken into account using the repeated statement in the MIXED procedure and an unstructured block diagonal residual covariance matrix. The model comprised the fixed effects of sampling time (home pen, shipment, lairage, slaughter), trial (2) and the two-way interaction sampling time x trial. To evaluate the effect of the repeated blood collection procedure on the concentrations of blood parameters at exsanguination the model considered the repeated blood collection act (yes or no), trial (2) and their two-way interactions as fixed effects. For the comparison of stress vocalization and lesions a generalized linear model were employed, applying the GLIMMIX procedure using a Poisson model (distribution = Poisson, link = log). For stress vocalization the model comprised the fixed effect time (loading, unloading, transportation, sampling) and for lesions the fixed effects time (home pen, after transport), trial (2) and group (treatment, control) and their interactions. From the data of IGF-system, for each animal the ratio of IGFBP-3/-2, the total amount of IGFBPs (indicator for IGF-binding capacity), the ratio of IGF-1/total IGFBPs (indicator for IGF-1 bioavailability) and the ratio of IGF-1 to IGFBP-2,-3 and -5 was calculated. Effects and differences were considered significant if p < 0.05. Data, which were below the limit of quantification, were excluded from the statistical analyses.

## Electronic supplementary material

Supplementary Information
