# Supplementary material for: Interference of stress with the somatotropic axis in pigs – lights on new biomarkers
Source: Sci Rep. 2017 Sep 21;7:12055. doi: 10.1038/s41598-017-11521-5 (PMC5608691; doi:10.1038/s41598-017-11521-5)
Supplement: Supplementary file 1 — Supplementary Information [file 41598_2017_11521_MOESM1_ESM.doc]

**Interference of stress with the somatotropic axis in pigs**

**– lights on new biomarkers**

Elisa Wirthgen^#1,3^, Martin Kunze^#1^; Sébastien Goumon^#2^, Christina Walz^1^, Christine Höflich^3^, Marion Spitschak^1^, Julia Brenmoehl^1^, Ellen Kanitz^4^, Margret Tuchscherer^4^, Winfried Otten^4^, Ulrike Gimsa^4^, Peter Schön^4^, Christian Manteuffel^4^, Armin Tuchscherer^5^ , Ralf Pfuhl^6^, Cornelia C. Metges^7^, Bernd Stabenow^8^, Sandra Erdmann^9^, Kathleen Schluricke^1^, Luigi Faucitano^10*^, Andreas Hoeflich^1*^

Institutes of ^1^Genome Biology, ^4^Behavioural Physiology, ^5^Genetics and Biometry, ^6^Muscle Biology & Growth, ^7^Nutritional Physiology ‘Oskar Kellner’, ^8^Experimental Animal Facilities, Leibniz Institute for Farm Animal Biology (FBN), Dummerstorf, Germany

^2^Institute of Animal Science, Department of Ethology, Prague, Czech Republic

^3^Ligandis GbR, 18276 Gülzow-Prüzen, Germany

^9^Fleischwerk EDEKA Nord GmbH, 19246 Lüttow-Valluhn, Germany

^10^Sherbrooke Research and Development Centre, Sherbrooke, Canada

**Suppl. Table 1** Effects of sampling time on the ratio of IGF-1 to IGFBP-3, IGF-1 to IGFBP-2, IGF-1 to IGFBP-5, and IGFBP-3 to IGFBP-2. Data are presented as LS-means ± SE and Tukey-Kramer procedure was used for pairwise comparisons. Significant differences are indicated by different letters.

| **ratio** | **Sampling time** | | | | | | | |
| --- | --- | --- | --- | --- | --- | --- | --- | --- |
|  | **home pen** | | **shipment** | | **lairage** | | **slaughter** | |
| **IGF-1/IGFBP-3** | 0.07 | ± 0.006**a** | 0.05 | ± 0.002**b** | 0.06 | ± 0.005 | 0.06 | ± 0.006 |
| **IGF-1/IGFBP-2** | 0.25 | ± 0.054 | 0.16 | ± 0.023 | 0.20 | ± 0.048 | 0.19 | ± 0.028 |
| **IGF-1/IGFBP-5** | 0.39 | ± 0.045**a** | 0.15 | ± 0.024**b** | 0.41 | ± 0.066 | 0.19 | ± 0.051**b** |
| **IGFBP-3/IGFBP-2** | 3.51 | ± 0.368 | 3.51 | ± 0.391 | 3.49 | ± 0.395 | 3.55 | ± 0.379 |

**
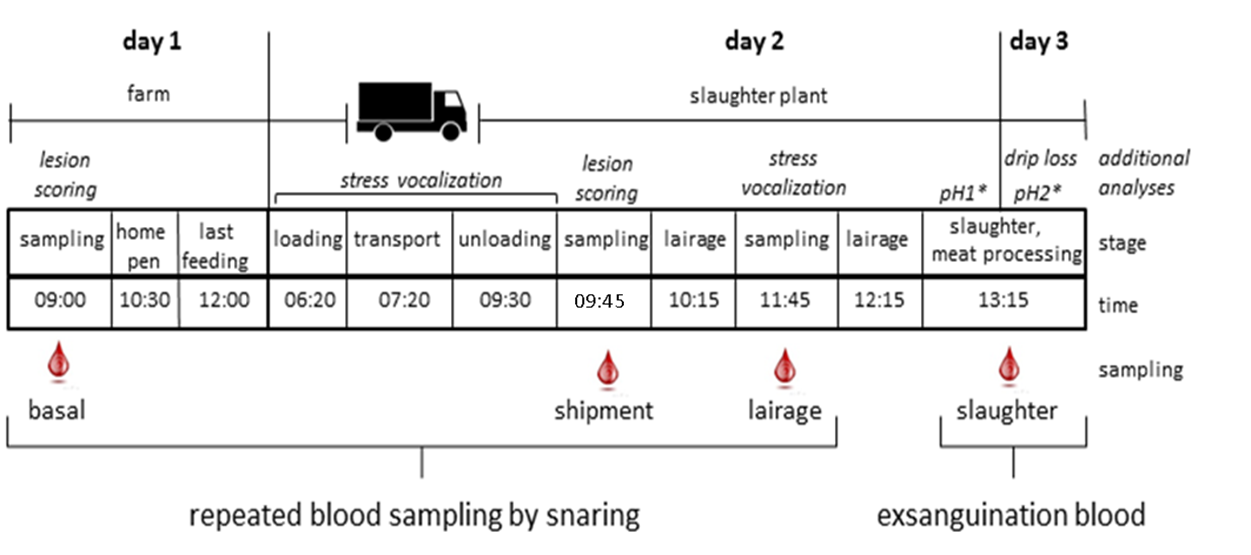
**

**Suppl. Figure 1** Experimental design. The animals were grouped at least 4 months before shipment. Group structure was maintained during the whole experimental procedure on farm, truck and slaughterhouse. Pigs were withdrawn of feed for 18 h (since 12.00 pm day 1) and loaded at the farm by a trained crew using boards. On arrival at the slaughterhouse, pigs were unloaded through a plane ramp using boards. Pigs were kept in lairage for 3 h and had free access to water and water sprinkling during that time. For blood sampling pigs were separated in 2 pens (2.5 m^2^/pig) and control pigs were separated in a third pen (1.25 m^2^/pig).


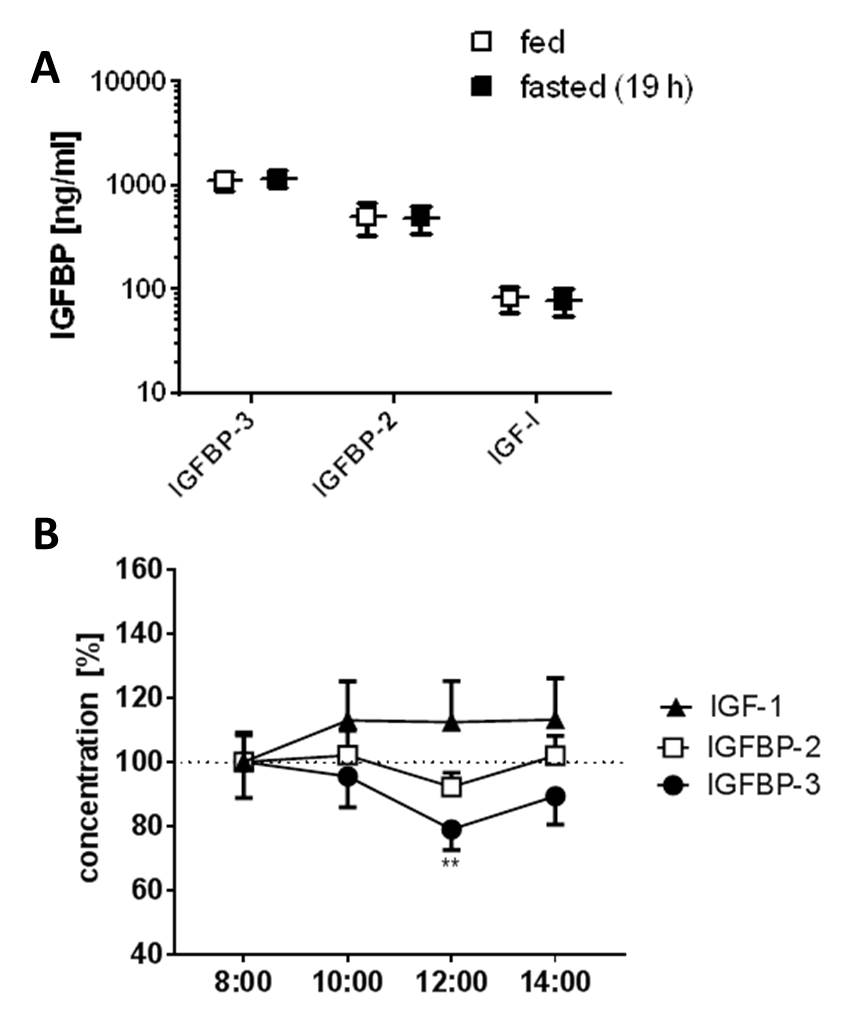


**Suppl. Figure 2** Effects of food removal (A) and time of day (B) on serum concentrations of IGF-1 and IGFBP-2 or -3 in pigs. Food was removed for a period of 19 hours. Diurnal blood collections were drawn between 8 a.m. and 2 p.m. n=7. **P<0.01

**
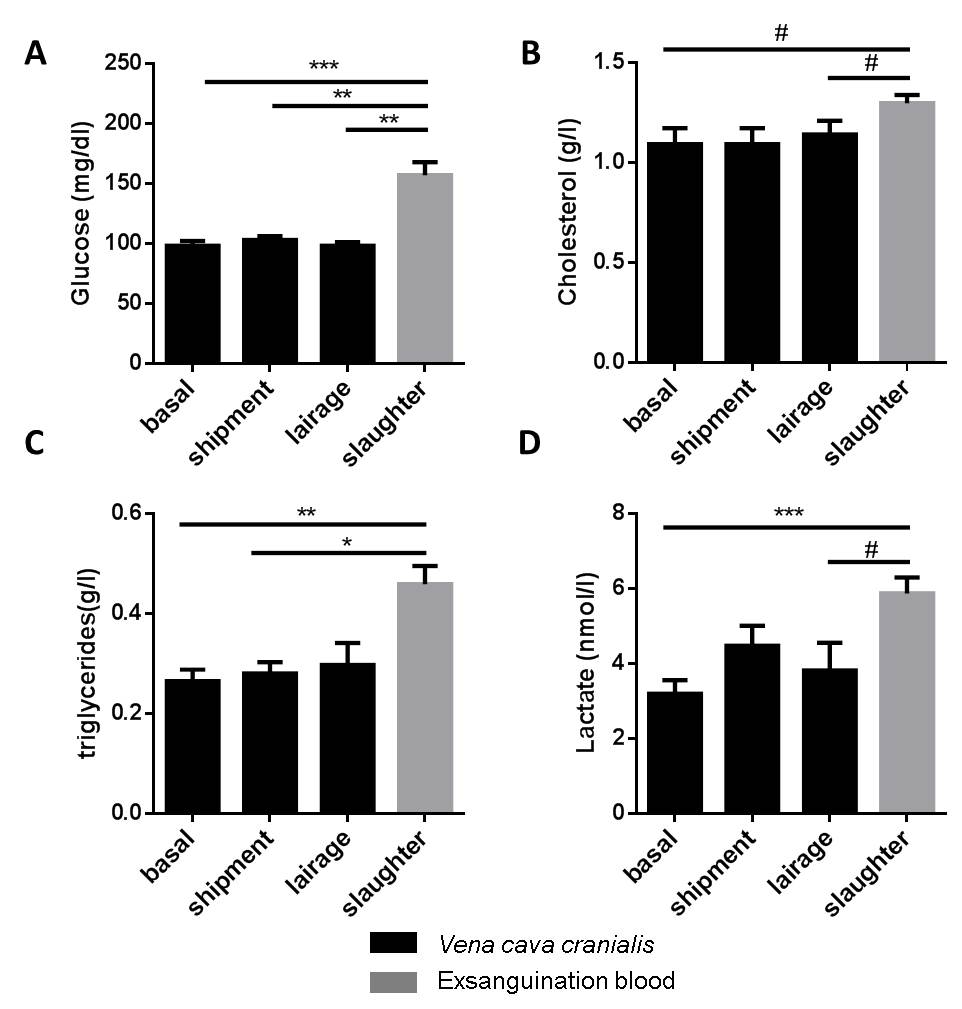
**

**Suppl. Figure 3** Parameters of energy metabolism at different sampling times of commercial transportation procedure. Plasma concentrations of Glucose (A), Cholesterol (B), Triglycerides (C) and Lactate (D) analyzed with enzymatic spectrophotometric assays. Data are presented as LS-means + SE. Lactate: n = 31 per sampling time. Glucose, Cholesterol, Triglycerides: n = 13 per sampling time. #p < 0.1, *p < 0.05, ** p < 0.01, ***p < 0.001
